# Would You Use It With a Seal of Approval? Important Attributes of 2,4-Dinitrophenol (2,4-DNP) as a Hypothetical Pharmaceutical Product

**DOI:** 10.3389/fpsyt.2018.00124

**Published:** 2018-04-20

**Authors:** Emma E. Bleasdale, Sam N. Thrower, Andrea Petróczi

**Affiliations:** ^1^Faculty of Science, Engineering and Computing, School of Life Sciences, Pharmacy and Chemistry, Kingston University London, Kingston upon Thames, United Kingdom; ^2^School of Sport, Exercise and Health Sciences, Loughborough University, Loughborough, United Kingdom

**Keywords:** diet pill, fat burner, 2,4-dinitrophenol, DNP, weight loss, bodybuilding, eating disorder

## Abstract

**Background:**

2,4-Dinitrophenol (2,4-DNP) is an effective but highly dangerous fat burner, not licensed for human consumption. Death cases reported for 2,4-DNP overdose, particularly among young adults, have raised concerns about the ineffective regulatory control, lack of education and risks associated with impurity, and the unknown concentration of 2,4-DNP purchased on the Internet.

**Methods:**

Using a sequential mixed method design and based on a hypothetical scenario as if 2,4-DNP was a licensed pharmaceutical drug, first we conducted a qualitative study to explore what product attributes people consider when buying a weight-loss aid. Focus group interviews with six females and three males (mean age = 21.6 ± 1.8 years) were audiorecorded, transcribed verbatim, and subjected to thematic analysis. Sixteen attributes were identified for the Best–Worst Scale (BWS) in the quantitative survey with 106 participants (64% female, mean age = 27.1 ± 11.9 years), focusing on 2,4-DNP. Demographics, weight satisfaction, and risk for eating disorder data were collected.

**Results:**

In contrast to experienced users such as bodybuilders, our study participants approached 2,4-DNP cautiously. Attributes of 2,4-DNP as a hypothetical weight-loss drug comprised a range of desirable and avoidable features. Of the 16 selected attributes, BWS suggested that long-term side effects were the most and branding was the least important attribute. Effectiveness and short-term side effects were also essential. Those in the >25 year group showed least concerns for legality. Neutral BWS scores for cost, treatment, degree of lifestyle changes required, and specificity required for the hypothetical weight-loss drug to be effective were likely caused by disagreement about their importance among the participants, not indifference.

**Conclusion:**

With advances in research, 2,4-DNP as a pharmaceutical drug in the future for treating neurodegenerative diseases and potentially for weight loss is not inconceivable. Caution is warranted for interpreting the BWS scores. Owing to the difference in what data represent at individual vs. population levels, with pooled data, the method correctly identifies attributes by which most people are satisfied but misrepresents attributes that are individually very important but not universally agreed. Whilst this may be an advantage in marketing applications, it limits the utility of BWS as a research tool.

## Introduction

Due to increased concerns of body weight and image, along with the widespread use of the Internet and social media platforms, the already considerable market of weight-loss drugs and supplements grows rapidly (1, 2). Within this market, various products are available which include substances that could pose health hazards to users. Like any other market where effective regulatory control is lacking (3), the supplement market is also open to unethical practice whereby products could contain unlicensed ingredients (4, 5) contaminated with controlled substances and/or deliberately spiked with potent controlled substances to increase effectiveness [e.g., Ref. (6–12)]. The adulterants in the latter being unconventional and dose set to produce the desired effect, deliberate dietary supplement fraud poses greater health risks than trace contamination from lack of quality control (13). In addition, regulatory effort to curb economically motivated fraud is further challenged by the readily available cross-border retail options on the Internet (14). Drugs that are withdrawn before marketing and thus not licensed for human consumption such as melanotan II or Cardarine (also known as Endurobol or GW-501516) (15–17) and/or reintroduced after being officially withdrawn decades ago [e.g., Ref. (18, 19)] remain available on the black market and easily obtainable *via* the Internet.

### Renaissance of 2,4-DNP

One example of substances not licensed for human consumption is 2,4-dinitrophenol (2,4-DNP), which is an effective but highly dangerous fat burner. Currently, 2,4-DNP has industrial use but it is not licensed for human consumption and its sale as such is prohibited around the world. Despite the danger, 2,4-DNP has re-emerged within the bodybuilding community and extreme dieters, particularly among young adults.

#### History of 2,4-DNP as a Weight-Loss Drug

The attractiveness of 2,4-DNP arises from the fat-burning effects without the need of dietary control (20). 2,4-DNP was used in diet pills for obesity treatment between 1933 and 1938 under brand names of Dinitriso, Nitromet, Dinitrenal, and Alpha Dinitrophenol. Owing to its severe side effects, diet pills containing 2,4-DNP were withdrawn from the market in 1938 (21, 22). Over 100,000 people were prescribed the drug, with claims of increasing metabolism by up to 50% at a harmless dose (21, 22). However, it was disputed whether the drug was as effective and harmless as evidence suggested; alongside DNP’s release to the public, warnings of the potential toxicity of the compound were issued by the council on Pharmacy and Chemistry (23). In 1938, there was enough evidence collected to suggest that DNP had potential lethal adverse effects and posed a threat to public health. As a result, 2,4-DNP was subsequently banned by the Federal Food, Drug, and Cosmetic Act (24). Efforts of regulatory bodies to protect the public from harm associated with 2,4-DNP are counterbalanced by the ease of access and availability through Internet retailers (2, 19), and by a plethora of online discussion forums that share experiences among users and readily offer guidance and advice on what and how to use to chemically boost athletic performance or to achieve the desired appearance (25).

#### Function

**Figure 1:**
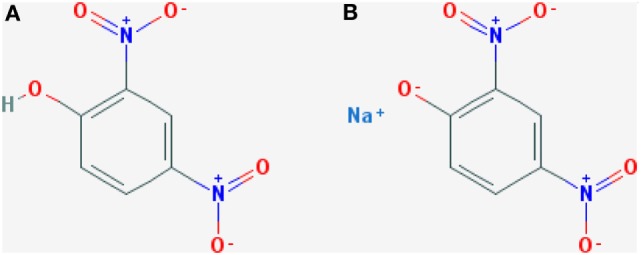
Chemical structure of 2,4-DNP produced in two forms: **(A)** 2,4-Dinitrophenol and **(B)** sodium dinitrophenolate. Taken from Petróczi et al. (19).

2,4-DNP is an organic compound which is chemically manufactured in two forms (see Figure 1). The product is the result from the hydrolysis of 1-chloro-2,4-dinitrobenze (26). The compound was initially used to manufacture products such as explosives and dyes. It was later discovered that it behaved as a protonophore in the human body which allowed the movement of protons from the mitochondrial intermembrane space across the inner mitochondrial membrane, acting as a strong uncoupler within oxidative phosphorylation (27).

The outcome of this process causes the breakdown of carbohydrates and fats (21). This allows energy from cellular respiration to be released as heat rather than being stored as adenosine triphosphate, resulting in reduced fat stores and a rise in body temperature (27). This was highly attractive as there was no recorded effect on proteins or nitrogen excretion (21). Past research has proposed the argument that DNP is ineffective, with some evidence suggesting that DNP has little effect on the rate of weight loss at a therapeutic dose in comparison to dietary restriction (28). In some cases, DNP at a therapeutic level only increased metabolic rate by <15% with an outcome of no weight loss, with some patients gaining weight due to increased appetite (29).

Recently, the novel central anti-obesity mechanism of the action of 2,4-DNP has been evidenced which offers new avenues for targeting obesity with 2,4-DNP and other mitochondrial uncoupling agents (30–32). The emerging evidence has suggested that mitochondrial uncoupling proteins (UCPs) have key roles in neuronal plasticity and resistance to metabolic and oxidative stress. UCPs are induced by activities such as caloric restriction and exercise. 2,4-DNP recreates a similar pathway to these activities. The resulting outcome has the potential to protect neurons against dysfunction and degeneration. This may lead to a possible product containing low doses of 2,4-DNP to improve diseases such as Alzheimer’s and Parkinson’s disease. A major concern would be to certify that the low dose of 2,4-DNP was therapeutic (33). Notably, research in these areas is primarily driven by finding new ways of targeting mitochondrial coupling and uncoupling, in which 2,4-DNP is used as a proof-of-concept drug rather than repurposing 2,4-DNP specifically.

#### Toxicity

These adverse side effects ranged from short-term nausea, vomiting, and increased pulse rate, to increasingly severe long-term effects such as cataracts, hepatotoxicity, and death (34, 35). The issue surrounding DNP is that the adverse side effects may be triggered at various doses, whether considered therapeutic or not. This is because the tolerance to the drug is different for each individual; therefore, doses that may be considered “safe” for some may be lethal to others. There has also been evidence of a person’s tolerance altering over time, allowing previously tolerated doses to become lethal (36).

#### Access

As 2,4-DNP is currently illegal for human consumption, there is concern over those accessing the drug whilst it is unregulated. Although manufacturers have the potential to contaminate or be inconsistent with the purity of their product, increasing its potential to cause harm, research has shown that this is not a major concern (19).

#### Motivation

2,4-DNP is used knowingly and willingly by obese individuals (37) and those desiring extreme outcomes, such as achieving unnaturally low body fat in bodybuilding (38), compensating for overeating among bulimia sufferers (39) or motivated by a rapid and effective weight loss and used 2,4-DNP as shortcut (19, 40). The drug is often taken in cycles, and information regarding dosage and personal programs is readily made available by bodybuilders for others in the industry to follow (25). Although this is concerning, bodybuilders appear to control the dose and follow a program to reduce the potential lethal effects (19). However, there is increasing concern of usage within vulnerable groups such as those with eating disorders or young and naïve users who are not fully informed about the drug. People’s willingness to take the risk with 2,4-DNP—whilst remaining low among the general population—has shown to increase considerably if there is a relatively large amount of weight gain within a short period of time (40).

#### Regulatory Efforts

Until recently when 2,4-DNP resurfaced in the gym-going population as a potent fat burner and more attention has been drawn to this substance, thanks to numerous media reports of DNP overdose deaths, law enforcement and regulatory bodies were largely unaware that protecting the public by controlling sales and access in the Internet era is an impossible task (19). Because 2,4-DNP—and many similar drugs—is easily available on the uncontrollable global online drug market, efforts of protecting the public are more likely to be effective if preventive and harm-reduction measures target the potential users, not solely focusing on controlling sales and the suppliers.

In fact, having dedicated agencies [FDA in the US, Food Standard Agency (FSA) in the UK, EFSA at the EU level] and regulatory policies in place means that perceived safety could backfire. Consumers of dietary supplements may make their decision in a false sense of safety about the products. In fact, research has shown that a significant proportion of physically active people rely on label information when making decision about supplements but only half were concerned about the quality of the information (41). It is recognized that providing more information does not automatically lead to a greater decision-making autonomy or better behavioral choices (42), nor are people sufficiently health-literate to be able to make the right decision amidst the complexity of evidence for pros and cons (43). Furthermore, preliminary results, with regard to 2,4-dinitrophenol (DNP—used as fat burner weight-loss product), suggest that willingness is inversely related with the severity of the health consequences (40). As such, willingness to take risks with DNP despite health warnings appears to be influenced more by the desired goal (the magnitude of weight people wished to lose) and having past experience with similar products than general risk-taking propensity or other psychosocial factors.

Due to the market increase *via* the internet, cases of 2,4-DNP misuse are being seen globally in countries such as America, England, and China (20, 44). The FSA has had to communicate repeatedly to publicly raise awareness about the potentially lethal and permanent effects of 2,4-DNP, in an attempt to combat the rising health complications and deaths caused by the drug by reducing those interested in the drug, alongside warning the current users.

Alarmingly, the plethora of personal accounts of successful 2,4-DNP use in bodybuilding websites, discussion boards, and forums (25) gives some the untrue impression that 2,4-DNP is suitable for human consumption and may be considered a “safe” product if used “properly.” Discounting the idea that 2,4-DNP poses danger because it may be disguised within slimming pills, research has shown that customers buy the product knowingly (19). However, 2,4-DNP poses serious health risks to new or naïve users who are not experienced with performance- and image-enhancing drugs or conscientious of dosage, and lack of knowledge on the extent of potential harmful effects (19). This has contributed to an increasing number of mortalities caused by 2,4-DNP (5, 37, 39, 44–46). It is also important to note that although isolated cases of treatments for 2,4-DNP overdoses have been reported with varying degrees of effectiveness [see (47)], there is to date no established cure or treatment for a 2,4-DNP overdose.

Looking forward, the future of 2,4-DNP as a pharmaceutical drug is not inconceivable. Although the use of 2,4-DNP is controversial, there might be a use for 2,4-DNP for treating extremely overweight or morbidly obese individuals in a clinically controlled environment.

The benefits in entertaining such hypothetical case are twofold: 2,4-DNP is an effective compound for weight loss and its use under a controlled clinical setting would require appropriate understanding of the unique toxicity profile of 2,4-DNP and training for clinical staff. The latter then would carry over to successfully treating accidental overdose and preventing tragic deaths. Media reports and medical case studies fail to portray how 2,4-DNP users feel and think about the drug. With a few exceptions [e.g., Ref. (19, 25, 40, 48)], thought processes and rationalization of 2,4-DNP use have not been adequately captured. Understanding people’s motives for taking risk with 2,4-DNP as well as understanding how they negotiate the risks and where they draw the line is vital for devising meaningful prevention and harm-reduction strategies [see Presentation S1 in Supplementary Material for an example of a real-world case study (49)].

### AIMS

One concern around the re-emerged 2,4-DNP is that despite official warnings and media reports, 2,4-DNP is continuing to show activity within the weight-loss community and it seems to be increasing in popularity. One plausible approach to mitigate risks against the unintended overdose is to produce 2,4-DNP as a weight-loss drug again to ensure quality control and safety of this—otherwise still dangerous—drug. This controversial proposition could be rationalized on the premise that regulating production and distribution, while still making the drug available, could reduce harm from questionable quality and uncertainty around concentration. Equally, it can be argued that this approach would still be too dangerous, and instead regulatory efforts should focus on improving control over the illegal supply. Regardless of the route taken for regulation, education with harm prevention and reduction in mind is warranted, and the more we understand the motives and barriers behind using 2,4-DNP (and similarly risky substances) the better we can tailor health education to the users’ wants, needs, and motives. Often, successful health education for prevention and harm reduction requires taking a holistic approach and addressing the problem in a broader context. In the present study, the key research question is not restricted to 2,4-DNP specifically, but rather, 2,4-DNP is used as a controversial example.

Thus, using a hypothetical scenario, in this study we set out to explore whether producing the drug with proper quality control and advice on safe use would (1) increase willingness to use and (2) reduce harm from 2,4-DNP and investigated what factors people would consider important in buying 2,4-DNP if it would be a licensed pharmaceutical drug.

Alongside this, we also investigated whether demographic details (age, gender, educational level) and health condition (disordered eating) influence the importance of these factors. This will give an indication of who may be at a higher risk of purchasing unsafe weight-loss substances such as 2,4-DNP.

## Materials and Methods

Based on the exploratory nature of the research questions, a sequential mixed method design (50) was used within the current study. Specifically, the research undertook two phases: first, we conducted two focus group interviews which served as an elicitation for the survey content, followed by a quantitative survey. Focus groups concentrated on the factors young people would consider before buying weight-loss supplements and drugs, such as 2,4-DNP. The results from the elicitation phase contributed toward a self-reported survey which was composed of 31 closed questions and investigated what factors would be considered most or least important in the possible scenario of 2,4-DNP as a licensed weight-loss drug.

### Ethical Considerations

The study was approved by the Research Ethics Committee of the Faculty of Science, Computing and Engineering, Kingston University, under the delegated approval scheme. Participation in the study was voluntary and anonymous. Participants were fully informed about the aim of the study and conditions of participation. Consent was implied by voluntary participation in the focus group and/or by completing and returning the survey. Focus group participants also gave written informed consent to the use of their demographic information such as age and gender, purpose for weight loss and past experience with weight-loss products—with anonymity preserved—for scientific purposes and academic dissemination. Consent was obtained prior to the focus group, and participants were asked to complete the “personal information sheet” which contained questions about the above demographic details. Participants received no compensation.

### Qualitative Phase: Focus Group Interviews

#### Participants and Sampling

Following institutional ethical approval, convenience sampling was used to recruit (in person) university students between 18 and 30 years of age *via* personal networks. The focus groups contained six females and three males with a mean age of 21.56 ± 1.71 years. In the focus groups, only two participants had previously used weight-loss substances both of which were female and with their main concern being appearance (see Table 1).

**Table 1:** Focus groups demographics.

Focus group	Gender	Age	Purpose for weight loss	Experience with weight-loss substances in the past
Group 1 M1	Male	23	Fitness/sport	No
Group 1 F1	Female	20	Appearance	Yes
Group 1 F2	Female	21	Appearance	Yes
Group 1 F3	Female	25	Appearance	No
Group 1 F4	Female	23	Health	No
Group 2 M1	Male	21	N/A	No
Group 2 M2	Male	21	Fitness/sport	No
Group 2 F1	Female	21	Fitness/sport	No
Group 2 F2	Female	19	Fitness/sport	No

The age distribution in the first focus group was slightly more spread (ranging from 20 to 25 years) than in the second group (age range of 19–21 years). Both groups were mixed in terms of gender and involvement in sport and exercise but only the first group included participants with the experience of using a weight-loss product.

#### Process

Participants were given documents providing a brief background on the topic (participants’ prior knowledge of 2,4-DNP was not assessed in the current study), a consent form, and a personal information sheet to gather the demographics of the focus groups (i.e., gender, age, purpose for weight loss, and whether participants had taken weight-loss substances—see Table 1). Participants were informed about the purpose of the study, the voluntary nature of participation, and the confidential nature of the focus groups. Focus groups lasted between 30 and 45 minutes were audiorecorded and transcribed verbatim by the first author.

#### Focus Groups

Semi-structured focus groups were used to collect information on topics surrounding weight-loss drugs and substances in terms of possible benefits, negatives, outcomes and specifically the factors considered when buying weight-loss drugs such as 2,4-DNP. Based on recommendations within the literature (51), each focus group consisted of four to five participants. Participants were asked to consider the scenario of 2,4-DNP as a possible weight-loss drug and what factors they or others may consider before buying the products. The focus group interview matrix, alongside the questions, is presented in Presentation S2 in Supplementary Material.

#### Data Analysis

Focus group transcripts were analysed using a thematic analysis. Following Braun and Clarke’s (52) procedures, transcripts were read and re-read to promote content familiarity. Data were then analysed *via* a process of line-by-line coding to allow themes (i.e., the factors considered when buying weight-loss drugs such as 2,4-DNP) to emerge. Once identified, themes were labelled and grouped together to create higher-order themes. Finally, the data were revisited to ensure that each theme was appropriately represented. Following the second focus group, a satisfactory level of saturation regarding important attributes of 2,4-DNP was reached.

### Quantitative Phase: Questionnaire Study

#### Participants and Recruitment

In line with the qualitative phase, individuals over 18 years of age were recruited for the survey phase using convenience and snowballing sampling techniques. Apart from the age limit of 18 and over, no specific inclusion/exclusion criteria were set for this phase of the research. The questionnaire was made available online using a closed survey platform (SurveyMonkey) and as a hard paper copy. The content of the two surveys was identical. This allowed participants to be recruited online *via* social media and in person.

#### Measures

##### Attributes of 2,4-DNP as a Hypothetical Weight-Loss Drug

Desirable attributes of a 2,4-DNP as a hypothetical weight-loss drug were identified using the Best–Worst Scale (BWS) technique which involves choice modelling. In this method, multiple options are provided in several iterations but only the best and the worst option are selected in each case. This method is a multiple-choice extension of the paired comparison method, which is scale-free and forces participants to make a selective choice among the issues under consideration (53). The attractiveness of the method in market research is that respondents are forced to trade off the most desirable features against the “would-be-nice-to-have” attributes, which resembles real-life decision making. For example, when having an ethically produced premium quality product at a low price is not possible, customers must make a choice of which attribute is more important to them (e.g., ethics, quality, or price). Similarly, an ideal weight-loss product would be highly effective but also pleasant and free of side effects but this may not be possible in real life.

In the current study, the BWS was formed around the 16 factors produced from the two focus groups (see Table 2). The template design was produced within Datagame (a gamification tool for online surveys, http://datagame.io/) (54) and was set to produce 20 unique sets of four of the 16 factors in each question without repeats. In the questionnaire, each attribute appears five times. In this survey, the BWS was embedded in a hypothetical scenario. The scenario provided a brief background of 2,4-DNP and its current use in society alongside its potential dangers. The hypothetical situation specified that a pharmaceutical company is considering reintroducing 2,4-DNP on the weight-loss drug market and want to explore what customers think about 2,4-DNP using a market survey. Participants are asked to place themselves as a participant in this market research and to consider what factors they felt were most or least important. The scenario and the full BWS survey are presented in Presentation S2 in Supplementary Material.

**Table 2:** Themes, theme explanations, and supporting evidence for the factors considered when buying weight-loss drugs such as 2,4-DNP.

Theme	Theme explanation	Supporting evidence
Accessibility	How easy is it to access the drug (e.g., online, pharmacy, prescription-only, over the counter)	“I think more people would be willing to buy it if it [2,4-DNP] were more readily available but I think for the sake of safety that you should buy it over the counter” (Focus Group 2—F1)

Effectiveness	How well the drug achieves the desired results (i.e., weight loss)	“I think if a drug is effective, and they are really good at marketing that, it would beat all the other factors, I mean you will always find a way to store it, or find a way to take it if it’s that important to you” (Focus Group 2—F1)

Degree of lifestyle change required	The extent to which individuals have to change their lifestyle for a drug to be effective (i.e., increasing exercise, water intake, managing diet)	“I definitely would want to do a bit of research, does it say it only works with something else, like do you have to drink a lot of water every day to make sure it works? Do you actually still have to do exercise? Or do you drop the weight by sitting on the sofa still?” (Focus Group 2—M2)

Adherence required	The period of time in which you have to take a drug for and the dose required	“If it’s something like weight loss, it’s gonna be quite a long time before you really notice a difference so having to remember to take it every 4–5 h would become a bit of a chore” (Focus Group 2—M2)

Dosage	How often a drug needs to be taken (e.g., once a week/several times a day)	“If it’s like a tablet, I have to take at a specific time of the day, even like several times a day, I’m not good at that so it would probably be like, maybe if like it was only effective if you like take it exactly at like 5 h intervals, then I would just be like that is never going to happen” (Focus Group 2—F1)

Short-term side effects	Temporary negative effects (e.g., headaches, mild rashes, pain) as a consequence of the drug	“If it’s small stuff you could live with like not driving a car, or even a rash as long as it’s not painful, people would be more inclined to just do it anyway…whereas if it’s something that actively stops you from doing something or causes a lot of pain and discomfort I think that’s when people would be like no, it’s not worth it” (Focus Group 2—M2)

Long-term side effects	Severe negative effects that become permanent/irreversible (e.g., chronic migraines, blindness) as a consequence of the drug	“I would be on board, if it was very temporary and I would lose weight as a result of it. I could use this drug for x amount of time, but I can’t do this, then I’d probably be willing to put my life on the side and lose weight, and then find my life again, but side effects that I would really would be a no for me, would be if I got really ill, or a danger that I become really ill… if I’m going to go blind or something” (Focus Group 2—F1)

Cost	The price of the drug	“I think the price would hinder me, as it [2,4-DNP] would probably cost ridiculous amounts of money. I think that would be at that point where I’d be like no I don’t want it that bad” (Focus Group 1—F2)

Formulation	The physical state of the drug (e.g., pill, liquid, powder form)	“I think there’s a lot of stigma around taking pills…like if you’re talking to someone and saying you’re taking pills for weight loss…their immediate reaction would be like are you sure, where did you get them from, are they legit kind of thing, I probably would go for a shake” (Focus Group 1—F2)

Specificity	If the drug targets a specific/localised area of the body or is generalised across the whole body	“Everyone has bits of their body where they have more fat than other parts of their body and if someone takes it and they start to lose weight like on their bum but not their stomach they might not take it anymore so, yeah, it [the drug] needs to target where” (Focus Group 2—M2)

Legality	Whether the use of a drug is within the law or not	“I think young people and athletes are the ones who are gonna buy something illegal… so, if someone just wants to lose a few kilos they’re not gonna look into anything illegal, I think they’ll just go to the pharmacy” (Focus Group 2—M1)

Reviews and experiences	Other people’s opinions and experiences of using a weight-loss drug.	“I have gone on the internet and researched so many things, drug control and stuff like that, and going on forums, other people’s experiences versus like science, and stuff like that helps, reading peoples experiences online and checking things, and obviously being careful and reading a tonne of things rather than just one website” (Focus Group 1—F3)

Branding	The extent to which a brand is known or recognisable for certain products	“I think it [branding] is important as not everyone knows a lot about drugs, so you just see brands and go “oh I’ve heard of that before” it must be better… I’ll probably buy whatever is more familiar even though it’s more expensive” (Focus Group 2—F2)

Interactions with other substances	If the drug interacts with and impacts on other medications or substances (i.e., stops other medication working)	“[You need to consider] other medications…because they can sometimes have like negative effects when drugs are combining with other drugs” (Focus Group 2—M2)

Treatment	How the drug is taken (e.g., orally, injections, suppositories)	“I don’t like putting things in water, it’s too much effort and it tastes horrible so as soon as I can take it in a tablet and it’s just done in like a couple of seconds, for me that’s ideal, it’d be things like powder or suppository that would be like a massive no” (Focus Group 2—F2)

Storage and preparation	How the drug needs to be stored (e.g., in the fridge) and prepared (e.g., needs to be dissolved)	“I’d probably be less inclined to take it, if it [storage and preparation] was complicated, yeah like if there was some sort of complicated process to it” (Focus Group 1—F2)

##### Eating Behaviour and Disordered Eating

To assess participants’ at risk status for disordered eating, six questions from the EAT-26 test [Part C (55)] were used. EAT-26 is an established screening measure (not a diagnostic tool) to determine a possible eating disorder or a person who may be at risk. Section C of the test comprises six questions: (1) *Gone on eating binges where you feel that you may not be able to stop?* (Defined as eating much more than most people would under the same circumstances and feeling that eating is out of control.); (2) *Ever made yourself sick (vomited) to control your weight or shape?* (3) *Ever used laxatives, diet pills, or diuretics (water pills) to control your weight or shape?* (4) *Exercised more than 60 min a day to lose or to control your weight?* (5) *Lost 20 pounds or more in the past 6 months?* (6) *Have you ever been treated for an eating disorder?* The first four questions were rated as Never/Once a month or less/two to three times a month/Once a week/two to six times a week/once a day or more whereas the last two questions were answered as Yes/No. The EAT-26, both the belief section and the behavioural aspects, is one of the most widely used screening tools for identifying high-risk individuals for referral to clinical evaluation, consistently showing good psychometric properties (56, 57). It has been noted that beliefs manifest to a larger extent than behavioural symptoms, suggesting that beliefs are the precursors for developing disordered eating (58, 59). Those who report behavioural symptoms respond to the belief items congruently, but the opposite is not necessarily the case (i.e., beliefs can present without behavioural symptoms).

##### Satisfaction With Weight

Satisfaction with weight was recorded with three progressive questions. First, participants were asked if they were happy with their current weight (Yes/No) and whether they wanted to lose weight (Yes/No). In case the answer was yes to the weight-loss goal, the main reason behind this goal was further explored. To facilitate statistical analysis, closed question format questions with pre-set answers were used (e.g., Appearance/Health/Fitness/Other, with open text option).

##### Demographics

Demographic information we collected included age, gender, ethnicity, employment status, and highest completed education level. The categories within the highest completed education level included GCSE, A-Level/B.Tech, Undergraduate level 4, Undergraduate level 5, Degree, Postgraduate, and other. Ethnicity categories were based on the categories recommended by the Office for National Statistics (60): White, Mixed/multiple ethnic groups, Asian/Asian British, Black/African/Caribbean/Black British, or other. Finally, employment status was split into five categories: unemployed, student, part-time, full-time, or other.

##### Data Analysis

Following the selection count method [e.g., used in Ref. (61–63)], we used simple count data analysis (i.e., the scoring was based on how many times an attribute is ranked as “most important” and “least important”). Previously, Marley and Louviere (64) showed that this simple calculation is a close and suitable approximation of the true scale values obtainable from multinomial logit analyses. When an attribute is selected as most important, a score of 1 was given, and when an attribute is selected as least important, a score of −1 is given. To obtain the BWS score for each item at the individual level, the difference between “most” and “least” rankings was taken, which resulted in a rank between +5 and −5. The aggregated BWS scores for the 16 attributes for the sample were obtained by calculating the average time that each attribute was mentioned. In this scale, a score of +5 indicates a “very important” attribute and a score of −5 indicates that the attribute is “not important at all.” By coding “least” important as a negative value and “most desired” as a positive value allows for calculating not only the preference counts but also to calculate the mean [and standard deviation (SD)] without the need to note whether the counts come from the most or the least preferred choices.

Descriptive data are reported as median, mean and SD, frequencies, and/or percentages. We dichotomised age (18–25 years and >25 years) and at-risk status for eating disorder (i.e., having at least two affirmative answers of the six screening questions). Two affirmative answers were used instead of the traditional “having an affirmative answer” because the relative high proportion of athletes in the sample might routinely control their weight for sport reasons. Comparisons between two groups were tested using a factorial ANOVA. The association between categorical variables was tested using chi-squared statistics with Fisher’s exact significance. Statistical significance was set at *p* < 0.05 and tested two-tailed unless specified otherwise. Excel version 2016 for windows and IBM SPSS Statistics 22 were used for data entry and statistical analysis.

## Results

### Qualitative Phase: Focus Group Interviews

Focus group interviews yielded 16 themes which reflected the characteristics and factors considered when buying weight-loss drugs such as 2,4-DNP. Themes, theme explanations, and supporting evidence are presented in Table 2. The themes on drug characteristics were used as attributes for the BWS in the survey.

It is important to note that despite an agreement in relation to the importance of drug characteristics, participants did not always agree on how important each attribute was or the reasons why they felt it was important. For instance, participants agreed that the cost of a drug (such as 2,4-DNP) was important but some participants felt that a high price point would prevent or discourage them from buying it. As one 21-year-old female with experience of using weight-loss substances explained: “*I think the price [of 2,4-DNP] would hinder me, as it would probably cost ridiculous amounts of money, I think that would be the point where I*’*d be like no I don*’*t want it that bad*” (Focus Group 1—F2). By contrast, other participants felt that the price would not prevent them from buying the drug and that they may actually choose a more expensive option, if they believed it would be more effective. As another 21-year-old female participant stated: “*I know myself if I went to buy a drug and there was one that cost 50p and one that cost £10, I would probably be like the £10 one is more effective*” (Focus Group 2—F1). The following quote captures these contracting views surrounding the importance of cost:

You get people that are like if it’s like £30 cheaper then its not going to make a huge amount of difference and you get people at the other end of the scale who go well it’s the most expensive so it must be the best. (Focus Group 1—M1)

In addition to cost, participants also differed in their views regarding the preferred administration (i.e., formulation) and how the drug is taken (i.e., treatment). For instance, some participants felt that there was a stigma associated with taking pills for weight loss: “*I think there*’*s a lot of stigma around taking pills though*…*like if you*’*re talking to someone and saying you*’*re taking pills for weight loss*…*their immediate reaction would be like are you sure, where did you get them from, are they legit kind of thing, I probably would go for a shake*” (Focus Group 1—F2), whilst other participants favoured the simplicity and efficiency of pills and favoured this over other formulations. The following quote from a 19-year-old female illustrates this point:

I don’t like putting things in water, it’s too much effort and it tastes horrible so as soon as I can take it in a tablet and its just done in a couple of seconds for me that is ideal, it would be things like powder and suppository that would be a massive no! (Focus Group 2—F2)

Building on this point, although the majority of participants preferred taking drugs orally (*via* pills or shakes), some participants felt that injecting drugs were favourable especially if it resulted in a reduced dosage. As one participant explained:

I would prefer an injection if it was less often… just because I wouldn’t have to remember every day, I would be happy to go to my doctor if it was like once a week… especially if you can just go to the nurse and like get it (Focus Group 2—F1)

### Quantitative Phase: Questionnaire

The survey sample consisted of 106 individuals (64% female). The mean age of the sample was 27.08 ± 11.92 years with the majority (*n* = 81) being between 18 and 25 years of age. For this reason, age groups were divided as 18–25 and over 25. Sixty-five percent of the participants were students.

#### Attributes of 2,4-DNP as a Hypothetical Weight-Loss Drug

**Figure 2:**
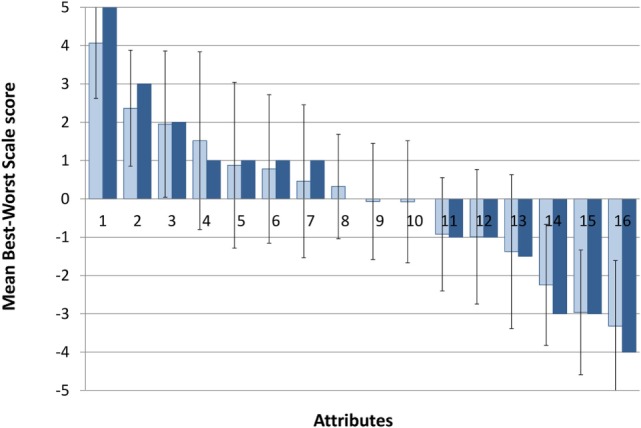
Aggregated Best–Worst Scale scores. Dark blue, median score; light blue, mean score; error bars represent standard deviation. Attributes on the *x*-axis are (1) long-term side effects, (2) effectiveness, (3) short-term side effects, (4) legality, (5) interactions with other substances, (6) reviews and experiences of others, (7) cost, (8) treatment, (9) degree of lifestyle change required, (10) specificity, (11) accessibility, (12) adherence required, (13) dosage, (14) formulation, (15) storage and preparation, and (16) branding.

The relative importance of the 16 attributes for 2,4-DNP as a hypothetical weight-loss drug is depicted in Figure 2 (depicting the average times an attribute was selected as most and least important) and Table 3 (summarising the outcome of the item count methods). Stratified analyses of the average times an attribute was selected as most and least by age group, gender, and at risk for disordered eating status are shown in Table 4.

**Table 3:** Attribute Best–Worst Scale counts, interval scale difference scores, and (pseudo-)ratio scale.

Attributes	Number of times ranked 5× as most important	Number of times ranked 5× as least important	Number of times ranked as most important (MI)	Number of times ranked as least important (LI)	Diff MI–LI	Rank (most important to least important)	Ratio ln√(MI/LI)
Accessibility	0	0	22	119	−97	11	−0.84
Adherence	0	1	132	31	−101	12	0.72
Branding	0	34	8	246	−238	14	−1.71
Cost	1	0	109	61	48	7	0.29
Dosage	1	1	30	180	−150	13	−0.90
Drug Specificity	6	0	60	68	−8	10	−0.06
**Effectiveness**	**0**	**4**	**252**	**6**	**246**	**2**	**1.87**
**Form**	**3**	**0**	**3**	**355**	−**352**	**16**	−**2.39**
Interactions (with medicines)	10	1	143	50	93	5	0.53
Legality	0	0	201	40	161	4	0.81
Lifestyle change	5	0	58	65	−7	9	−0.06
**Long-term (LT) side effects**	**0**	**0**	**439**	**2**	**437**	**1**	**2.70**
Reviews and experiences	0	0	132	50	82	6	0.49
Short-term (ST) side effects	5	0	226	21	205	3	1.19
**Storage and preparation**	**0**	**17**	**3**	**317**	−**314**	**15**	−**2.33**
Treatment	0	0	71	37	34	8	0.33

**Table 4:** Ranked means Best–Worst Scale attribute scores within 2,4-DNP scenario by age and gender.

Attributes	18–25 years male (*n* = 31)	18–25 years female (*n* = 46)	Over 25 years male (*n* = 7)	Over 25 years female (*n* = 22)	Not at risk for DE male (*n* = 29)	Not at risk for DE female (*n* = 39)	At risk for DE male (*n* = 9)	At risk for DE female (*n* = 29)
Accessibility	−1.23^a^	−1.20^a^	0.41^a^	−0.57^a^	−0.32	−0.89	−1.78	−1.00
Adherence	−0.90	−1.02	0.0	−1.36	−0.72	−0.89	−0.78	−1.45
Branding	−2.90^b^	−3.43^b^	−2.57^b^	−3.91^b^	−2.72	−4.03	−3.22	−3.00
Cost	0.61	0.64	−0.43	−0.09	0.97	0.26	−0.11	0.59
Dosage	−1.42	−1.13	−1.0	−1.95	−1.38	−1.56	−1.22	−1.17
Drug Specificity	−0.16	−0.33	−0.14	0.62	−0.07	0.14	−0.44	−0.24
Effectiveness	2.45	2.41	2.00	2.25	2.31	2.21	2.56	2.57
Form	−1.68	−2.63	−2.14	−2.27	−2.07^c^	−2.44^c^	−0.78^c^	−2.62^c^
Interactions with other substances	0.97	1.07	−0.14	0.82	0.72	1.03	0.88	0.93
Legality	0.68^d^	1.61^d^	2.57^d^	2.18^d^	1.00	1.97	1.11	1.55
Lifestyle change	−0.2	0.22	−1.14	−0.14	−0.50	0.11	0.00	0.10
Long-term (LT) side effects	3.68	4.07	4.14	4.59	3.59	4.21	4.33	4.28
Reviews and experiences	0.73	0.83	0.14	0.95	0.71	0.87	0.33	0.86
Short-term (ST) side effects	2.10	1.73	0.71	2.59	1.86	2.03	1.78	2.00
Storage and preparation	−2.74	−2.96	−2.43	−3.45	−2.59	−3.08	−3.00	−3.17
Treatment	0.35	0.43	−0.14	0.18	0.17	0.46	0.56	0.21

*Statistical significance (at *p* < 0.05) is marked*.

*^a^Main effect: age [*F*(1,101) = 13.957, *p* < 0.001]*.

*^b^Main effect: gender [*F*(1,102) = 5.080, *p* = 0.026]*.

*^c^Main effect: gender [*F*(1,102) = 10.267, *p* = 0.002]; interaction effect: [*F*(1,102) = 4.580, *p* = 0.035]*.

*^d^Main effect: age [*F*(1,102) = 4.883, *p* = 0.029]*.

Based on the survey results, the most important attributes for such a drug were long-term side effects, followed by effectiveness and short-term side effects, with branding, formulation, and route of administration (formulation) being the least important. Drug interactions, user reviews, cost, treatment, lifestyle changes required, drug target specificity, access, and duration of the treatment were placed in the middle region (BWS scores between +1 and −1).

**Figure 3:**
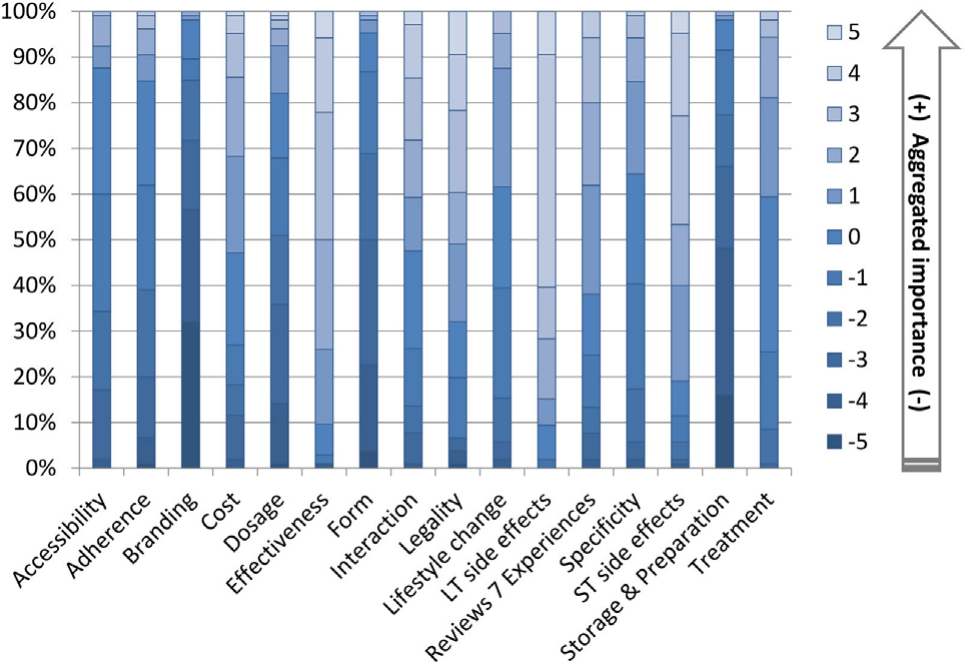
Best–Worst Scale choice counts [*n* (100%) = 106].

Table 4 and Figure 3 offer a more detailed analysis of the BWS choices. Taken together, the results indicated that across both gender age groups, the highest scoring factor was long-term side effects (LT side effects) and the lowest scoring factor was branding. The female group ages 18–25 also rated short-term side effects as important. Both younger age groups and females over 25 selected effectiveness as an important factor and storage and preparation as unimportant. The older age groups and females between 18 and 25 years of age ranked form as unimportant. The older age groups showed a higher concern in terms of legality, in comparison to 18–25 males and females. Statistically significant differences were only found for age effect on accessibility (*p* < 0.001) and legality (*p* = 0.029), and gender effect on branding (*p* = 0.026) and formulation (*p* = 0.002). The latter also showed a significant interaction effect between gender and at-risk status (*p* = 0.035). These are marked in Table 4, along with the corresponding test statistics.

In stratified analysis by reasons for weight loss, the data showed a statistically significant difference in ranking dosage and long-term side effects between those who were satisfied with weight and those who were not. Those satisfied with body weight scored dosage (*p* = 0.040) and long-term side effects (*p* = 0.019) with a significantly lower rank in comparison to those unsatisfied with body weight. Alongside this, there was a significant difference in long-term side effect ranks between those who want to lose weight and those that do not. Those wanting to lose weight scored a significantly higher importance (*p* = 0.003) towards long-term side effects than those who do not want to lose weight. The data show that those who selected health as the main reason for weight loss scored effectiveness differently from those who chose appearance (*p* = 0.014) or fitness (*p* = 0.012). Those who chose health for main reason for weight loss scored effectiveness significantly lower than those who chose the appearance or fitness. The only significant result within eating behaviour was those who were not at risk ranked branding significantly lower than those at risk.

**Figure 4:**
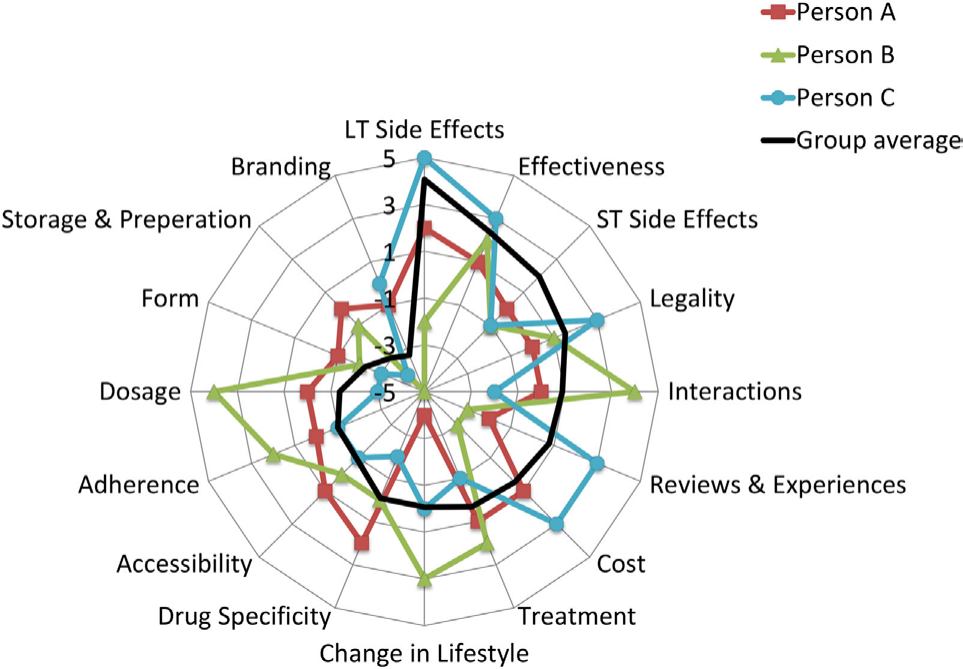
An illustrative example of personal preferences for attributes. Person A: a 21-year-old male who is happy with his current weight and he does not want to lose weight, at risk for disordered eating score is 1/6; Person B is an 18-year-old male who is happy with his current weight and he does not want to lose weight, at risk for disordered eating score is 0/6; Person C is a 20-year-old female, who is not happy with the current weight and she wants to lose weight, at risk for disordered eating score is 4/6. Black line represents the average BWS score for the group.

Juxtaposing qualitative and quantitative data, the overall picture emerges from the detailed analyses suggesting that attributes everyone agreed upon as important or as unimportant scored accordingly on the BWS scale (close to 5 and close to −5) but those attributes where participants in the focus group disagreed scored as “neutral” (neither important or unimportant). Using three participants from the set of 106, Figure 4 illustrates these notable differences at the individual level.

As the black line (representing the sample average) shows, the attributes were arranged in the order of importance, going from not important (−5) to very important (+5). Overlaying individual scores on the sample average highlight the contrasting views on attributes such as cost, change in lifestyle, and dosage. By contrast, the patterns of scores are fairly consistent across the participants for long-term health effects, effectiveness, and branding.

#### Eating Behaviour

The 18–25 age group showed a higher risk of developing a potential eating disorder than those aged over 25 years of age. Specifically, within the 18–25 age group, under 10% of males and 20% of females were classified as being a non-risk. By contrast, within the over 25-age group, over 30% were classified as a non-risk.

Participants who selected appearance or fitness for the purpose of weight loss are at a higher risk of a potential eating disorder than those who chose health. These results may show some bias, as only a small number (14% of overall response) of participants were represented within the health category.

#### Satisfaction With and Intention to Lose Weight

Satisfaction with weight was significantly associated with gender (χ^2^ = 6.076, *p* = 0.016) and age (χ^2^ = 6.114, *p* = 0.017). Satisfaction with current weight and “at-risk” status for developing eating disorder only reached the level of statistical significance if one-tailed test statistics were considered (χ^2^ = 3.992, *p* = 0.067/*p* = 0.036 one-tailed).

Intention to lose weight showed statistically significant association with gender (χ^2^ = 9.206, *p* = 0.003) but not age (χ^2^ = 1.718, *p* = 0.252). Similar to the satisfaction with current weight, “at-risk” status for developing an eating disorder only reached the level of statistical significance for the intention to lose weight if one-tailed test statistics were considered (χ^2^ = 4.402, *p* = 0.053/*p* = 0.028 one-tailed).

**Figure 5:**
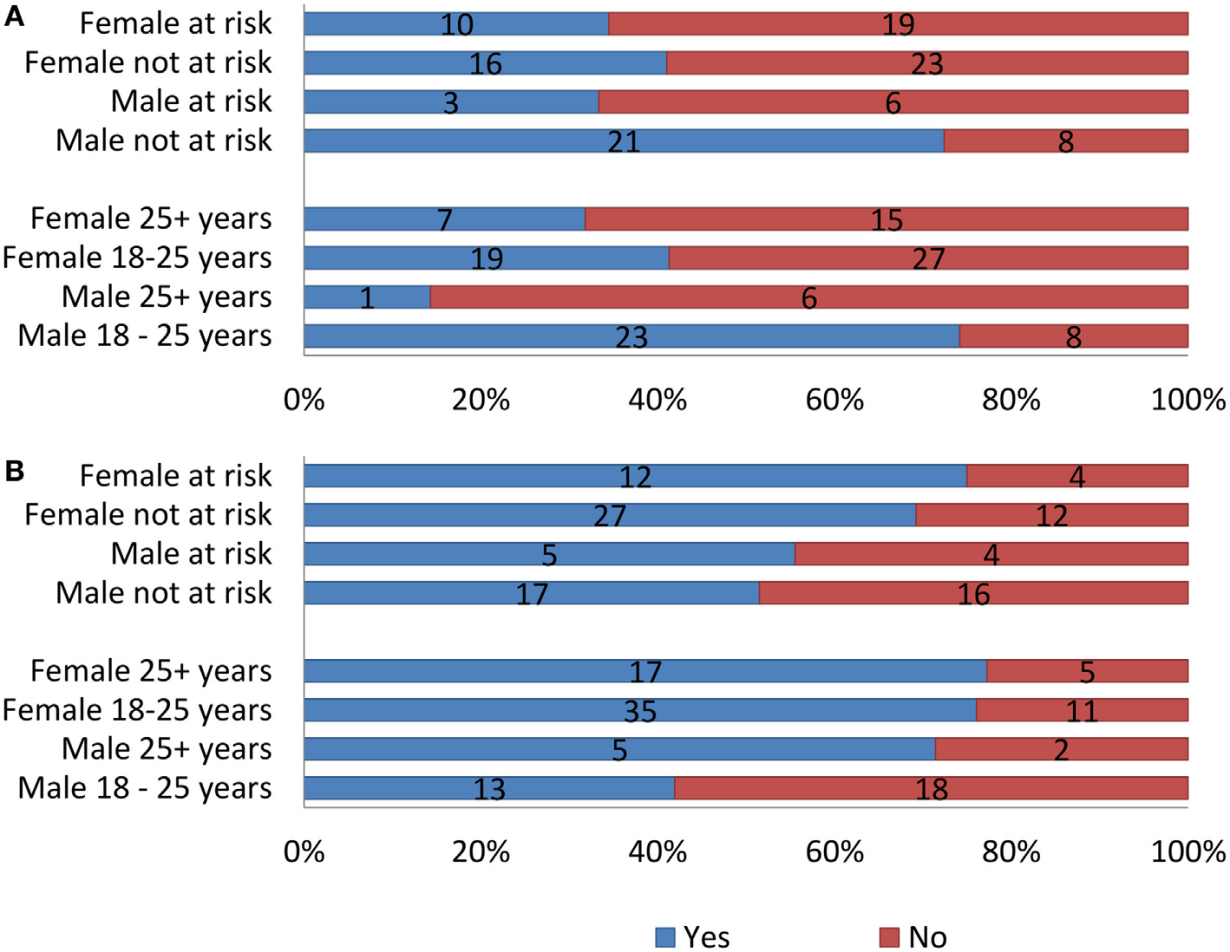
**(A)** Satisfaction with current weight. **(B)** Intention to lose weight in the stratified sample by age, gender, and at risk for developing eating disorder.

Using a stratified sample (Figure 5), the data indicate a significant dissatisfaction in weight from the over 25 age groups with 68.2% of females (25+ years) being unhappy with current weight and 6 of the 7 males over 25 years felt the same. Age played a significant role in weight satisfaction for males (χ^2^ = 8.808, *p* = 0.003). The 18–25 age groups indicated more weight satisfaction in comparison to the over 25 age group. The female 18–25 age group still indicated slight dissatisfaction, with 58.7% being unhappy with body weight, in contrast to 74.2% of males aged 18–25 showing satisfaction of weight. In terms of losing weight, over 70% of males and females over 25 along with females between 18 and 25 years of age expressed desire. By contrast, only 41.9% of males between 18 and 25 years of age wanted to lose weight.

The data showed a substantial difference in weight satisfaction in different ethnic groups. Those identified with white ethnic group were the most dissatisfied with their weight, with 57% being unhappy with their current weight. The mixed/multiple ethnic group showed a balanced response with a 50:50 ratio, whereas both the black/African/Caribbean/Black British and Asian/Asian British groups show less dissatisfaction with weight, with 66.7 (Black) and 77.8% (Asian) being satisfied with their current weight, respectively.

Stratifying the sample by “at-risk” status for disordered eating showed only one statistically significant association. Among males, at-risk status and weight satisfaction were associated (χ^2^ = 4.508, *p* = 0.037). Regardless of the at-risk status, 60–65% of the females were dissatisfied with their current weight and 70–75% wanted to lose weight. Over 70% of the at-risk male group was satisfied with their weight but 51.5% still wanted to lose weight.

#### Reason for Weight Loss

**Figure 6:**
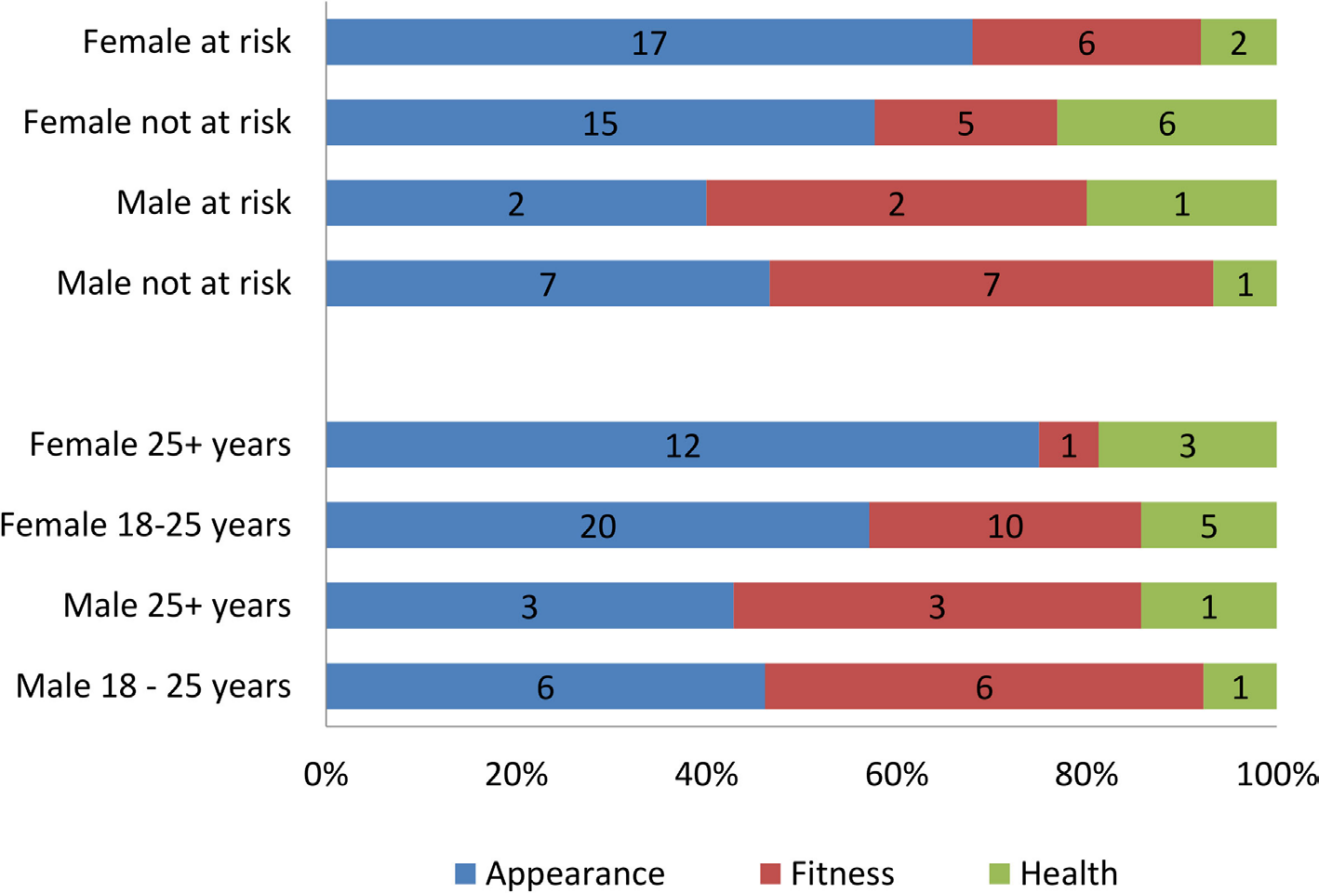
The main reason for wanting to lose weight in the stratified sample by age, gender, and at risk for developing eating disorder.

Age and at-risk status for developing eating disorder were independent of the reasons for weight loss. Gender, however, showed statistically significant association with reasons for weight loss (χ^2^ = 8.958, *p* = 0.031), with appearance dominating the reasons for females. Frequency counts in the stratified sample (Figure 6) show that the most prevalent reason for weight loss among females over 25 is appearance with 80% choosing this option. In females between 18 and 25 years of age, the results are more varied with appearance coming top (57.1%) followed by fitness (28.6%) and then by health (14.3%). In males aged 18–25, the most common reason for weight loss was fitness and appearance (3/7 both) followed by health (1/7). Males over 25 years of age showed a very similar pattern. Overall, women showed a greater concern towards appearance than men and men showed a greater concern over fitness than women.

Generally, at-risk status for both genders is manifested in similar patterns. Not-at-risk females indicated health reasons as most important for wanting to lose weight as opposed to those at risk who put appearance first. Among males, both “at-risk” and “not-at-risk” groups ranked appearance and fitness as equally important. There was no significant association between age, gender, or at-risk status.

## Discussion

2,4-DNP is present in the Internet black market and increasingly gaining attention, not only in the hard core bodybuilding community but also among the members of the general public. This project aimed to look into the factors people may consider before buying a weight-loss drug such as 2,4-DNP. In order to give an indication of who is more susceptible to buy 2,4-DNP and to inform public health policies, we also explored how the importance of these attributes was influenced by personal factors such as age, gender, weight satisfaction, and the desire to lose weight.

The overall results are in line with the previous study using a hypothetical scenario with 2,4-DNP (40). Participants in the study by Hoxha and Petróczi (40) were comparable in terms of age and body consciousness, and the study also evidenced inverse relationship between the severity of the health consequences (side effects) and willingness to take risks with 2,4-DNP as an unlicensed industry chemical. Our study indicated that this relationship is not affected by having a seal of approval for human consumption or quality-controlled pharmaceutical production, or branding. The effect of the drug on the body and lifestyle—both on the desirable and on the avoidable spectrum—appeared to be the most influential factor for considering 2,4-DNP.

Women showed a greater concern towards appearance than men but less concern over fitness. This is in line with the exiting literature which indicates that exposure to media images depicting a thin-ideal body type relates to body image concerns within women (65, 66). This may indicate why females are more concerned over body weight in accordance to appearance and explain the increasing number of deaths in young females due to black market slimming pills. Notably, however, 2,4-DNP users are predominantly males (19, 25) which might be due to the fact that 2,4-DNP is considered a controversial drug even within bodybuilding and among those who otherwise use a wide range of performance- and image-enhancing substances (19, 48).

In contrast to females, the male participants showed a greater concern towards fitness, especially within the younger 18–25 age group. There is increasing research indicating men and boys are becoming more concerned with body image and are undergoing more peer pressure to become slender and muscular [e.g., (67)]. This may be one reason why 2,4-DNP is prevalent within the bodybuilding community (48).

Notably, the approach to manage the risks with 2,4-DNP differed between the current study and of Hoxha and Petróczi (40), and the studies in which the primary focus was on bodybuilders’ lived experiences and rationalisations (19, 25, 48). Contrary to the general views about risks and unpleasant side effects expressed in the present study was avoidance (i.e., not doing it if ….), bodybuilders’ approach to 2,4-DNP-related risks was to have control over as many aspects as possible (19, 48). One plausible reason for the observed differences is the use of a situation, which does not translate directly to nor can be interpreted as actual behaviour. The other, more likely, reason is the qualitative difference in the target population. Bodybuilders approached 2,4-DNP from the position that using drugs to achieve the desired body shape is normal but 2,4-DNP is one extreme measure whereas members of the general public showed a conservative approach to using drugs in the first place.

Interestingly, having in-depth knowledge and understanding of the drug, which was pertinent in all bodybuilding-focused studies (19, 25, 48) did not feature among the attributes of 2,4-DNP as a hypothetical pharmaceutical drug although some reference was made to the mode of action in the focus groups.

### Attributes

The most common factors considered before buying weight-loss products were accessibility, reviews, experiences, adherence (course), treatment, short-term side effects, long-term side effects, effectiveness, storage, preparation, dosage, change in lifestyle, cost, interactions with other substances, drug specificity, legality, branding, and form of drug.

The highest and lowest ranking factors across the different age and gender groups were long-term side effects and branding. The long-term side effects were expected to receive a high mark of importance as people tend to avoid harm especially with the severity and length of effects being unknown. Both young (male and female) age groups and females over 25 years of age selected effectiveness as a very important factor, indicating that these groups may have a higher interest with weight loss.

The older age groups showed a higher concern in terms of legality in comparison to those who are younger. These findings suggest that younger people may be more willing to take risks to achieve their desired physique and as a result are more likely to buy illegal weight-loss drugs. This may go some way to explaining why the majority of mortalities due to 2,4-DNP are among young people (44).

Those wanting to lose weight scored significantly higher in terms of importance regarding long-term side effects than those who did not want to lose weight. Interestingly, this potentially contradicts other research as it may be assumed that those wanting to lose weight would have greater-risk willingness, so would see a reduced concern towards potential hazards. However, this potentially could be because those who wanted to lose weight related more to DNP scenarios, than to those who did not want to lose weight and may have given a more realistic consideration to the potential harms of weight-loss drugs.

Those who chose health as the main reason for weight loss scored effectiveness significantly lower. This might be because losing a significant amount of weight in a short period can be considered unhealthy. Therefore, those who are health conscious wouldn’t be as concerned with how effective the drug was. Also, it may be assumed that those choosing health over appearance and fitness are less likely to purchase a weight-loss product due to the potential hazards and so wouldn’t consider effectiveness as important as side effects.

Judging from the quantitative BWS scoring alone, it would appear that some attributes (e.g., cost, treatment, degree of lifestyle changes required, and specificity of the hypothetical weight-loss drug) are neither important nor unimportant when in fact any one of these factors alone would stop a person taking 2,4-DNP. The rationale for using BWS instead of scaled responses was to capture the relative importance of attributes if a risky substance such as 2,4-DNP would be manufactured and sold as a pharmaceutical product, and indirectly to shed the light on what aspects are the most important to potential consumers of 2,4-DNP. The latter would help to address public health concerns about 2,4-DNP and devise effective preventive and/or harm-reduction strategies.

Best–Worst Scale method (also known as maximum difference scaling) originates from consumer research exploring relative preferences. The BWS model is a multichoice extension of the scale-free paired comparison where respondents are not asked to assess the absolute importance of an issue on some arbitrary scale but presented as a trade-off choice (i.e., would you rather have option A or option B). Because of this characteristic, BWS is thought to resemble the actual cognitive process by which consumers make product choices (68). In subsequent applications, outside marketing showed that the BWS method adequately captures abstract values and value systems (61, 69–71) and is suitable to assess a wide range of issues such as health care (72, 73), education (74, 75), and sport (71, 76).

Since its conception more than 25 years ago, limitations of a direct preference assessment with BWS have started to emerge [e.g., 77, 78], showing a clear discrepancy between consumers’ declared relative importance of an attribute such as packaging or calorie information and the attributes’ actual influence on purchasing. This limitation, however, is not linked to the BWS but rather caused by the discrepancy between declared preferences and behaviour, the latter being influences by a host of other—temporary—factors. The issue our results highlighted is different and likely caused by the information process between individual vs. aggregated levels. BWS is thought to model the thought process of a single individual; thus, BWS score is reflective of what this person explicitly expresses for preference. When the data are aggregated across the sample to represent the population, individual differences are lost in the process because extreme polar views on the same attribute cancel each other out. The result of this process is a strong agreement about attributes that all individuals believe are important and attributes that are considered less important. Attributes that are in fact critically important but without agreement in how these attributes should manifest (e.g., formulation and route of administration of a drug in our study, or the level of lifestyle changes required to make the drug work) falsely manifest as neither important nor unimportant. From the practical point of view, BWS results could be quite useful for marketing purposes because it makes sense to provide features in a product that all individuals deem important and avoid those that are considered less important. However, this is a serious limitation for applying BWS as a research tool because the richness of the data is lost in translation from the individuals to the group. Similar caution has been made by Krucien et al. (79) finding that, in comparison with the discrete choice experiment (DCE) method, BWS method yields lower quality data for developing Health Utility Indices. Based on a systematic review, Whitty and Oliveira Gonçalves (80) suggest that profile-case BWS and DCE are equally robust measures but they might tap into different constructs. Multiprofile BWS might be concordant with DCE outcomes but this observation was based on a single study, thus requiring further verification. Unfortunately, these observations are not directly applicable to our present study because we used object-case BWS (81, 82). Object-case BWS, while successfully addressing some concerns associated with rating scales having ties (i.e., everything very important and socially desirable responding), BWS lacks accuracy and discriminatory power to compare respondents with differing preferences (81, 82). Furthermore, it must be noted that BWS scores alone do not offer any insight into how important the entire choice scenario to the respondent and how important each attribute is (81, 82). The combination of our quantitative and qualitative results offers support to this observation. In our study, we partially addressed the first aspect by including and analysing BWS in the context of weight satisfaction, weight-loss goals, and disordered eating. To address the latter aspect, an additional ranking scale is required prompting respondents to not only rank attributes in a forced choice setting but also evaluate the importance of each of them. Including this additional information could address the issue we highlighted, namely the misrepresentation of some attributes as “neutral” by including weighting to each attribute. Future studies using BWS for research are also recommended to incorporate methods that afford individual-level analyses. Triangulating the individual and aggregated BWS results with qualitative and/or behavioural data could provide useful insights into the method and facilitate further development.

### Limitations

The questionnaire consisted of 31 questions, with some sections such as the DNP scenario and BWS containing a considerable amount of information resulting in a lengthy survey. It was mentioned by a few participants that the survey was too long, with at least 26 participants taking over 10 min to complete the survey. This may have led to some questions being answered superficially, reducing the accuracy of the results.

The majority of participants were young females which inadvertently led to females aged between 18 and 25 years to be over-represented within the data. Given that females are more conscious about weight, talk more about weights (83), and more likely to use weight-management clinics and weight-loss products (84), this characteristic of the sample may not be a limitation but a true reflection of the weight-loss product market.

Lastly, the eating behaviour scale showed some discrepancies, as the tool defines a person at risk of an eating disorder with a score of 1 or higher. However, the survey collected information from a high proportion of young people, mainly students. In this population, many are highly active within sport and require high levels of exercise to control weight and remain competitive. Therefore, many athletes scored at least a 1 due to controlling their weight by exercise. Alongside these, many athletes may experience eating binges more frequently due to this high activity, in which their body needs to quickly replace depleted energy stores.

## Conclusion

Due to the range of side effects, which vary in severity depending on a person’s tolerance, 2,4-DNP has remained illegal for human consumption since its ban by the FDA in 1938. Facilitated by easy access to the substance *via* the Internet, legislation cannot curb its use by the general public which raises public health concerns. Despite numerous warnings to make the public aware of the dangers from using 2,4-DNP, the drug is still showing activity within the weight-loss community.

With advance in research, 2,4-DNP as a licensed pharmaceutical drug in the future for treating neurodegenerative diseases with chronic micro-dosing and potentially for aiding weight loss is not inconceivable. However, owing to the media reports of deaths and irresponsible marketing, supply, and use, 2,4-DNP has a reputation of being very risky and rightly so. Participants in this study exhibited a reassuringly cautious and conservative approach to a risky drug like 2,4-DNP. Focusing on young adults, we showed that those most interested in weight loss are females predominantly 18–25 years of age and indicated that both males and females under 25 years exhibited a higher risk for disordered eating. Due to the rising body pressure effects on these age groups and with a reduced concern towards legality, this group of young people are at risk of becoming susceptible to different weight-loss products, including 2,4-DNP. Vast differences in social group norms (e.g., bodybuilders, athletes) around using pharmaceutical aids to weight loss were noted. There is little doubt that the market for such products exists and current control policies are inadequate; thus there is a need for finding new ways for prevention and harm reduction. Failing to control the risk through supply and access, public health policies should consider pragmatic solutions for controlling 2,4-DNP-related harm *via* education as well as research into the possibility of making 2,4-DNP a safer drug by controlling purity and quality as well as efforts to mitigate against side effects.

However, our results expand beyond 2,4-DNP and speak for young adults’ approach to using pharmaceutical products for achieving a “desired” body, which provide useful insights for public health policies. Caution is warranted for interpreting the BWS scores. Our combined qualitative and quantitative results showed that the BWS method is capable of correctly identifying attributes most people feel the same way but misrepresents attributes that are individually very important but not agreed upon as unimportant or insignificant. This feature of the BWS method is very suitable for marketing purposes but outcomes should be interpreted cautiously in research applications.

## Ethics Statement

The study was approved by the Research Ethics Committee of the Faculty of Science, Computing and Engineering, Kingston University, under the delegated approval scheme. Participation in the study was voluntary and anonymous. Participants were fully informed about the aim of the study and conditions of participation. Consent was implied by voluntary participation in the focus group and/or by completing and returning the survey.

## Author Contributions

AP conceived the study and developed the research protocol with EB. EB collected data and analyzed the data with AP and ST. All authors contributed equally to drafting the manuscript and have read and approved the final version.

## Conflict of Interest Statement

The authors declare that the research was conducted in the absence of any commercial or financial relationships that could be construed as a potential conflict of interest.
