# Supplementary material for: Would You Use It With a Seal of Approval? Important Attributes of 2,4-Dinitrophenol (2,4-DNP) as a Hypothetical Pharmaceutical Product
Source: Front Psychiatry. 2018 Apr 20;9:124. doi: 10.3389/fpsyt.2018.00124 (PMC5919945; doi:10.3389/fpsyt.2018.00124)
Supplement: Supplementary file 1 [file Presentation_1.PDF]

APPENDIX

Interview matrix and questions (guide)

Weight loss substance use:

- What - sort of substances are used; what benefits may you/others expect?
- Where – can these substances be accessed?
- When – would you take them?
- How - do they work; are they taken; long would you use them?
- Why – do people consider/use these substances?

Attitudes towards medication:

- Is there a preference in how you take medication e.g. pill/ injection: Preferences in form - comparison to previously used medications?
- If orally, is there preference in accordance to appearance (size, shape, colour, coating)?
- What are your thoughts on the adherence of a drug; do you think the public could struggle- if so why? – explain adherence if needed.
- Do you turn to generic drugs or are you more inclined to certain brands? - Branding
- Would it be a concern to be taking multiple medications? -Multiple medications
- Do you think you or others have a specific area for weight loss or is it more general? Target of weight loss – specificity of drug
- What sort of effects have/would you expect from drugs for weight loss? Side-effects – long term; short term

Topics:

- Preferences in form - comparison to previously used medications
- Adherence
- Branding
- Multiple medications
- Target of weight loss – specificity of drug
- Side-effects – long term; short term

2,4-DNP as hypothetical weight-loss drug: The Best-Worst Scaling (BWS) Question

Scenario: 2,4-Dinitrophenol (2,4-DNP) is a man-made drug, which was used in 1930s to aid weight loss. It proved to be very effective, however, was later discovered to cause severe side effects depending on a person's tolerance, which varied between each individual. This led to its ban from human consumption by the FDA in 1938. However, 2,4-DNP is still available on the market for use as an antiseptic and as a pesticide. This has led to its increasing popularity for use within weight loss after its ban among bodybuilders and those with disordered eating. Following a few fatal incidents, the Food Standard Agency issued a warning about 2,4-DNP in 2012. Although 2,4-DNP is not licensed

for human consumption and it is illegal to sell for such purposes, the substance is easily accessible on the Internet retail sites and is used - often with considered safety measures - by bodybuilders. 2,4-DNP is dangerous but a very effective weight-loss promoter with numerous temporary (lethargy, excessive sweating, severe headache, allergic reactions, bloating) and potential for chronic (cataracts) side effects, including death if overdosed.

Imagine the following hypothetical scenario: Because of its effectiveness, pharmaceutical companies have a renewed interest in 2,4-DNP. They consider re-introducing 2,4-DNP to the diet-pill / weight-loss drug market. In order to develop a business case, they need to explore what the potential customers think about 2,4-DNP and launch a market survey. Imagine that you are invited to participate and asked to answer a set of questions about 2,4-DNP as a weight-loss promoter. There is no right or wrong answer. It is your honest opinion that matters.

If you were to use 2,4-DNP, which factors would you consider as most and least important.

There are 16 factors to consider. Below are the meanings behind each factor. Please read them very carefully before you start making your selections.

1. ***Accessibility*** - how easy it is to access the drug (e.g., online, pharmacy, prescription-only, over-the counter, supermarket, drugstores, etc.)
2. ***Effectiveness*** - how well the drug achieves the desired results (results being weight loss)
3. ***Change in life style*** - having to make lifestyle changes alongside drug use, so drug is effective e.g. increasing exercise/ water intake, managing diet
4. ***Course/Adherence*** - the degree to which a patient correctly follows medical advice (e.g., following the course of the drug through a long period of time and maintaining the right dose - therefor not forgetting drug dose/ increasing dose due to missing dose or wanting to increase results)
5. ***Dosage*** - how many times drug is needed to be taken/ frequency of drug (e.g., once a week/ five time a day)
6. ***Short Term side effects*** - temporary negative effects (e.g., headaches, mild rash, minor pain)
7. ***Long Term side effects*** - more sever negative effects that may become permanent/irreversible (e.g., chronic migraines, blindness)
8. ***Cost*** - refers to the price of an item (drug)
9. ***Form*** - refers to the state of the drug (e.g., pill form, liquid or powdered)
10. ***Drug Specificity*** – refers to where the drug targets e.g. if it effects (weight loss) in a localised area or generalised
11. ***Legality*** – the quality or state of being in accordance with the law
12. ***Reviews & Experiences*** – other people’s opinions
13. ***Branding*** - refers to known products (e.g., Nurofen for ibuprofen)
14. ***Interactions with Other Substances*** - if the drug interacts with other medications or substances - stops medication working, e.g., birth control
15. ***Treatment*** – how the drug is taken (e.g., orally, injections, suppositories)
16. ***Storage & Preparation*** – (e.g., stored in the fridge, needs to be dissolved).

Which of these attributes would you consider the MOST and the LEAST important out of the various combinations? Please select only one attribute as most important and only one attribute as least important in each set. There are 20 sets to answer in total. Each attribute appears five times.

Least important (select only one)	ATTRIBUTES	Most important (select only one)
☐	Dosage	☐
☐	Change in Lifestyle	☐
☐	Long Term Side Effects	☐
☐	Effectiveness	☐
☐	Storage & Preparation	☐
☐	Short Term Side Effects	☐
☐	Treatment	☐
☐	Accessibility	☐
☐	Adherence (course)	☐
☐	Reviews & Experiences	☐
☐	Cost	☐
☐	Interactions with Other Substances	☐
☐	Drug Specificity	☐
☐	Legality	☐
☐	Branding	☐
☐	Form	☐
☐	Adherence (course)	☐
☐	Accessibility	☐
☐	Long Term Side Effects	☐
☐	Drug Specificity	☐
☐	Interactions With Other Substances	☐
☐	Short Term Side Effects	☐
☐	Legality	☐
☐	Dosage	☐
☐	Form	☐
☐	Cost	☐
☐	Change in Lifestyle	☐
☐	Storage & Preparation	☐
☐	Branding	☐
☐	Reviews & Experiences	☐
☐	Effectiveness	☐
☐	Treatment	☐

☐	Accessibility	☐
☐	Reviews & Experiences	☐
☐	Form	☐
☐	Dosage	☐
☐	Adherence (course)	☐
☐	Treatment	☐
☐	Legality	☐
☐	Change in Lifestyle	☐
☐	Drug Specificity	☐
☐	Storage & Preparation	☐
☐	Effectiveness	☐
☐	Interactions with Other Substances	☐
☐	Branding	☐
☐	Short Term Side Effects	☐
☐	Cost	☐
☐	Long Term Side Effects	☐
☐	Short Term Side Effects	☐
☐	Adherence (course)	☐
☐	Effectiveness	☐
☐	Form	☐
☐	Accessibility	☐
☐	Interactions with Other Substances	☐
☐	Branding	☐
☐	Change in lifestyle	☐
☐	Legality	☐
☐	Long Term Side Effects	☐
☐	Storage & Preparation	☐
☐	Reviews & Experiences	☐
☐	Treatment	☐
☐	Cost	☐
☐	Drug Specificity	☐
☐	Dosage	☐
☐	Long Term Side Effects	☐
☐	Treatment	☐
☐	Form	☐
☐	Interactions with Other Substances	☐

☐	Legality	☐
☐	Cost	☐
☐	Effectiveness	☐
☐	Accessibility	☐
☐	Drug Specificity	☐
☐	Short Term Side Effects	☐
☐	Reviews & Experiences	☐
☐	Change in Lifestyle	☐
☐	Adherence (course)	☐
☐	Storage & Preparation	☐
☐	Dosage	☐
☐	Branding	☐
