# Supplementary material for: Would You Use It With a Seal of Approval? Important Attributes of 2,4-Dinitrophenol (2,4-DNP) as a Hypothetical Pharmaceutical Product
Source: Front Psychiatry. 2018 Apr 20;9:124. doi: 10.3389/fpsyt.2018.00124 (PMC5919945; doi:10.3389/fpsyt.2018.00124)
Supplement: Supplementary file 2 [file Presentation_2.PDF]

‘Rita’ is 28 years old. She has grown up with two brothers who were really keen on bodybuilding. […]

Rita came in contact with bodybuilding drugs in the gym. […] Rita also wanted to lose some fat and asked about specific substances that might help with it. Someone at the gym recommended DNP.

At first, Rita was sceptical about it. She turned to the Internet to get more information. When she recalls what she was thinking she asks: “*Who wouldn't be sceptical? DNP was described like a serious drug that kills you instantly.. but after seeing the guy during and after cycle still alive and fine; and then seeing other bodybuilders taking it and not only do fine but getting good results. I wanted the fat loss quickly. If you ask anyone who trains, I think they would do it as well.*”

At the end, Rita decided to take DNP and T3. She got them from the Internet but she admits that she had no idea if the pills she got were the real thing. Reflecting on her experiences, Rita says that, at first, taking DNP was not very dramatic and she felt that it did not do much at all; but the second week was very hard. She asked one of her brothers to stay with her for the second week. Rita admits that “*first he told me off for taking that stuff... but he was sort of curious too and he helped.*” Recalling her experiences, Rita continues: “*I was sweating so much, and then I was getting cramp in my muscles, insomnia at night, lethargy during the day. That was the worst thing during work out. If I drank water, I got that horrible bloated feeling.*”

Also, Rita understands that no drugs do magic. “*You have to control your lifestyle and you have to understand what those substances do to you*” - she says, then adds – “*You should only take DNP if you are serious about your workout regime. There is no point in taking it and going through all that hassle and lose the fat to just gain it back because your diet is poor.... Your knowledge about general health has to be there. It would be much easier doing a liposuction if you don't actually want to work for your goal.”*

Rita also talks at length about the importance of a supportive broader environment. Other gym users and the person who sold the DNP to her explained all the side effects and helped her to use it safely. She says that it was “actually really nice of them to be honest as really they didn't have to, they could have just sold it to me and that's it.” Rita argues that the problem is not with the drugs per se, but the users. In her opinion, it is really important to be prudent and respect the drug. She believes that there is a need for reliable advice and guidance to help those who want to use substances and she feels lucky that her environment provided guidance for her journey.

Taken from the Safe You project: https://issuu.com/safeyou/docs/full_digital, pp 63-65; Illustration: Umberto Pezzini, Togada Studio, used with permission. The case study is also available in video format on YouTube: https://www.youtube.com/watch?v=OghX8D-xSp8.
